# Robust Encoding in the Human Auditory Brainstem: Use It or Lose It?

**DOI:** 10.3389/fnins.2015.00451

**Published:** 2015-12-01

**Authors:** Alexandre Lehmann, Erika Skoe

**Affiliations:** ^1^Department of Otolaryngology Head and Neck Surgery, McGill UniversityMontreal, QC, Canada; ^2^International Laboratory for Brain, Music and Sound Research, Center for Research on Brain, Language and MusicMontreal, QC, Canada; ^3^Department of Speech, Language, and Hearing Sciences, Cognitive Science Program Affiliate, University of ConnecticutStorrs, CT, USA

**Keywords:** auditory brainstem, plasticity, music, amusia, tone-deafness

The human auditory brainstem faithfully represents the acoustic structure of sounds (Galbraith et al., 1995). In musicians, presumably because music training and exposure places high demands on the auditory system, brainstem encoding of both speech and musical sounds is more robust than in non-musicians. It is believed that the corticofugal system, a vast network of efferent connections within the auditory neuroaxis, drives top-down plastic changes underlying the enhancements in brainstem encoding in musicians and other populations with fine-tuned auditory abilities (Wong et al., 2007). Could these same mechanisms lead to impoverished brainstem encoding in those individuals whose musical skill proficiency is below average? Two recent studies on brainstem encoding in amusia, a congenital music disorder, shed a complementary light on this issue (Lehmann et al., 2015; Liu et al., 2015). Together they suggest that a subcortical deficit in auditory processing, which varies as a function of the degree of musical (dis)ability, can emerge as a consequence of limited meaningful interactions with music. We discuss how this notion of auditory detuning fits with the emerging view on top-down induced subcortical plasticity in auditory processing and address its implications for auditory rehabilitation.

Musicians are a paradigmatic example of individuals who have benefitted from their interactions with an acoustically enriched environment. From a neural standpoint, lifelong musical training has been linked with enhanced cortical mechanisms of sensory-motor processing (Münte et al., 2002; Herholz and Zatorre, 2012) and more robust auditory brainstem encoding (Musacchia et al., 2007; Parbery-Clark et al., 2012; Skoe and Kraus, 2013; Strait and Kraus, 2014; Weiss and Bidelman, 2015), even in the face of sensorineural hearing loss (Parbery-Clark et al., 2013) and aging (Parbery-Clark et al., 2011; Bidelman and Alain, 2015). Moreover, emerging behavioral research suggests that musicians with dyslexia might be spared from auditory disabilities associated with dyslexia (Bishop-Liebler et al., 2014; Weiss et al., 2014), providing further support for the claim that enriched musical experience has a beneficial influence on the auditory system by endowing greater resilience when the auditory system is compromised by auditory processing disorders, sensorineural taxation/decline, aging, or disease (Skoe and Kraus, 2014). Similar sensory benefits are purported for bilinguals and tonal language speakers (Krishnan et al., 2005, 2010a; Krizman et al., 2012, 2015). For musicians, training appears to generalize beyond the domain of training (i.e., music), as sensory enhancements are found in the brainstem response to both music and speech stimuli in individuals partaking in lifelong (Musacchia et al., 2007) or short-term musical training (Tierney et al., 2013). This generalization has been theorized to result from shared neural resources for speech and music at a subcortical level (Patel, 2014).

An unstated assumption in the literature on experience-dependent plasticity of the subcortical auditory system is that there is some basic, though ideosyncratic, congenital capacity to encode sound that can be sharpened with extensive exposure to enriched soundscapes. Conversely, might this basic capacity to encode sound erode if the auditory system is not stimulated by an acoustically-diverse landscape? Here we argue that the same mechanisms that enable the subcortical auditory system to develop more acute abilities in the face of enriched auditory exposure also make it more permeable to impoverished environments throughout life.

When deprived of rich auditory input during development, the auditory system becomes more broadly tuned, as shown in animal models (e.g., Zhang et al., 2001, 2002; Oliver et al., 2011). However, comparatively little is known about how acoustically-limited sound environments compromises auditory acuity in humans with normal hearing. Recent work suggests that children from socio-economically depressed homes have weaker auditory brainstem encoding, possibly as a consequence of reduced exposure to rich auditory input and increased exposure to noise (Skoe and Kraus, 2013; Krizman et al., 2015). In human adults, temporary sound deprivation (monaural ear plugging), has been shown to result in rapid adaptive plasticity of the middle-ear reflex, a mechanism mediated by the brainstem (Munro and Blount, 2009); yet the long term consequences of this deprivation are unknown.

While sensory detuning may be a consequence of deprivation, other evidence suggests that in non-deprived environments, sensory tuning, and detuning go hand in hand. For example, developmental studies suggest that infants undergo a perceptual narrowing in the first year of life, during which they show increased sensitivity to native speech sounds and decreased sensitivity to non-native sounds (to which they have had little to no exposure) (Werker and Tees, 2005; Kuhl et al., 2006). This balance of tuning-detuning likely reflects a more general phenomenon that occurs throughout the lifespan whereby listeners become optimally tuned to the recurrent sounds of their environment, at the expense of acuity to uncommon/infrequent sounds. For instance, Zheng (2012) reported that adult rats exposed to noisy environments lost auditory sensitivity in quiet environments but achieved better perceptual abilities in noise than the control group. Other evidence for environmental fine-tuning of the auditory system comes from adult speakers of tonal languages and adult musicians with different musical histories. Adult tonal language speakers have heightened brainstem encoding of native tonal contours but not non-native contours (Krishnan et al., 2010b). However, current evidence suggests that newborns from tonal language-speaking families do not differ from newborns from English-speaking families on these same brainstem encoding measures (Jeng et al., 2011), suggesting that sensory tuning to one's native language emerges between infancy and adulthood. In line with the work on tonal language speakers, Strait and colleagues reported increased brainstem sensitivity in adult musicians hearing the sound of their primary vs. non-primary musical instrument (Strait et al., 2012). Drawing on these findings, we now propose that a musical disability might detune the very same mechanisms that are tuned by enriched exposure to music.

Two recent studies of auditory encoding in amusia, a congenital disorder of music perception (Peretz, 2013), provide support for the possibility that the human auditory brainstem undergoes a loss of sensory acuity as a consequence of a musical disorder. We recently compared auditory brainstem encoding in a group of adults with amusia relative to a control group, matched in terms of age, sex, years of education, and audiometric function (Lehmann et al., 2015). We found evidence of an auditory impairment in the amusics compared to the controls; notably, the impairments mirrored advantages found in musicians, suggesting that musical impairments can weaken the aspects of sound encoding that are strengthened through musical training. Whereas musicians have faster brainstem responses—in which the stimulus harmonics are more robustly encoded compared to non-musicians—we found that amusics had delayed responses and less robust harmonic encoding than non-musician controls. Across the population of amusics and controls, we also found that worse musical abilities, as assessed by the Montreal Battery for the Evaluation of Amusia, were associated with greater neural delays and greater reductions in harmonic encoding, suggesting that individual differences in musical ability occurring within the general population may be reflected in how the auditory brainstem encodes sound. In contrast to our findings, a second recent study on amusia (Liu et al., 2015), found no evidence of brainstem encoding abnormalities to speech or music in an amusic group that differed from our population both with respect to age (their population was on average 40 years younger) and language background (Cantonese vs. French). We offer three not-mutually exclusive explanations for how auditory impairments might have manifested in one group of amusics but not the other.

The first explanation is that the younger Cantonese group does not have abnormal auditory encoding to either speech or music because their extensive experience with pitch in the context of a tonal language has bolstered their auditory systems, allowing them to overcome or be spared from the brainstem impairments associated with their musical disability (cf., Skoe and Kraus, 2011, 2014; Bishop-Liebler et al., 2014; Weiss et al., 2014). A second explanation is that brainstem abnormalities associated with musical deficits are not congenital but require that the auditory system undergo restricted interactions with music for an extended duration and that the Cantonese group has not met this critical duration. This explanation, while appealing in the current context, does not have strong empirical support. Lastly, if, as reported in a series of recent studies, musical experience can lessen age-related changes to the auditory system (Zendel and Alain, 2012; Parbery-Clark et al., 2013; Bidelman and Alain, 2015), this sets up the possibility that deprived exposure to music over one's lifetime might have the opposite effect by inducing rapid or more premature aging.

At their core, all three proposed mechanisms share a common “use it, or lose it” principle. However, we are not proposing that auditory brainstem function can be completely lost; the obligatory nature of auditory brainstem processes suggests that this is impossible. Instead we propose that limited musical exposure can weaken the auditory system within certain physiological limits. Another underlying facet of all three mechanisms is that auditory brainstem impairments associated with amusia are not the cause but the consequence of amusia. Whereas the literature on congenital amusia points to it being a disorder that primarily affects higher-level, association areas of the brain (Peretz et al., 2002; Hyde et al., 2011), recent evidence suggests anomalies earlier in the processing stream (Albouy et al., 2013). We propose that atypical higher-level cortical responses to music may over time backpropagate to weaken sensory encoding, resulting in subtle impairments in early cortical potentials (Albouy et al., 2013) and progressive detuning of the auditory brainstem via corticofugal processes. In support of this idea, musical training has been shown to strengthen efferent connections within the auditory system down to the level of the cochlea (Perrot and Collet, 2014) suggesting that limited exposure to music and/or aberrant cortical processes might weaken these very same connections.

If use-dependent principles govern the auditory brainstem, this raises several questions: Can this principle be observed in the general population and what aspects of auditory exposure are critical to the detuning of the brainstem? How does this detuning manifest in other auditory disorders? For instance, would beat-deafness—the rhythmic counterpart of amusia—selectively impact timing, and no other aspects of sound encoding? What is the relative role of age vs. tone-language expertise in the putative protective effects observed in the Hong Kong study (but not ours)? Assessing both young and aging amusic populations who speak tonal or non-tonal languages will shed further light on this issue. Longitudinal studies are also needed to elucidate the time course of the mechanism that sharpens or detunes the brainstem response and more clearly dissect the relative contribution of cortical structures in driving this type of plasticity.

If limited exposure to music listening or making can have negative effects, this would support the idea that music has a role in rehabilitation and in education more generally. Recent studies suggest that music training accelerates neuro-development in children (Skoe and Kraus, 2013) and adolescents (Tierney et al., 2015), and staves off the aging process (Zendel and Alain, 2012; Parbery-Clark et al., 2013; Bidelman and Alain, 2015). Together with the outcomes of our recent study, this suggests that musical activities (performance, listening, etc.) provide a fertile training ground for exercising the auditory system and promoting cognitive development and function over the lifespan. Similarly, such training could benefit other populations that undergo auditory rehabilitation, especially those who face perceptual challenges with speech training or fail to comply with other rehabilitation methods (Sweetow and Henderson Sabes, 2010). For instance, deaf individuals whose hearing has been restored with a cochlear implant struggle to perceive speech in noise and prosody. Because both capacities are known to improve with musical training, such training could help those individuals overcome these impairments (Chen et al., 2010; Fu and Galvin, 2012; Patel, 2014). In addition, our findings suggest that musical activities might provide a beneficial method for keeping the aging auditory system “in shape.”

In summary, the inherent plasticity of the auditory system primes it for being sensitive to the acoustic environment in a way that can either positively or negatively affect how sound is encoded subcortically. We believe that experience-dependent detuning of the auditory system may explain variations in auditory acuity in both typical and atypical populations, making it a topic ripe for future investigations.

## Conflict of interest statement

The authors declare that the research was conducted in the absence of any commercial or financial relationships that could be construed as a potential conflict of interest.
